# Ultrasensitive Label-Free Sensing of IL-6 Based on PASE Functionalized Carbon Nanotube Micro-Arrays with RNA-Aptamers as Molecular Recognition Elements

**DOI:** 10.3390/bios7020017

**Published:** 2017-04-17

**Authors:** Farhad Khosravi, Seyed Masoud Loeian, Balaji Panchapakesan

**Affiliations:** Small Systems Laboratory, Department of Mechanical Engineering, Worcester Polytechnic Institute, Worcester, MA 01532, USA; fkhosravi@wpi.edu (F.K.); smloeian@wpi.edu (S.M.L.)

**Keywords:** Carbon Nanotube Biosensors, IL6, Field Effect Transistors

## Abstract

This study demonstrates the rapid and label-free detection of Interleukin-6 (IL-6) using carbon nanotube micro-arrays with aptamer as the molecular recognition element. Single wall carbon nanotubes micro-arrays biosensors were manufactured using photo-lithography, metal deposition, and etching techniques. Nanotube biosensors were functionalized with 1-Pyrenebutanoic Acid Succinimidyl Ester (PASE) conjugated IL-6 aptamers. Real time response of the sensor conductance was monitored with increasing concentration of IL-6 (1 pg/mL to 10 ng/mL), exposure to the sensing surface in buffer solution, and clinically relevant spiked blood samples. Non-specific Bovine Serum Albumin (BSA), PBS samples, and anti-IgG functionalized devices gave similar signatures in the real time conductance versus time experiments with no significant change in sensor signal. Exposure of the aptamer functionalized nanotube surface to IL-6 decreased the conductance with increasing concentration of IL-6. Experiments based on field effect transistor arrays suggested shift in drain current versus gate voltage for 1 pg and 1 ng of IL-6 exposure. Non-specific BSA did not produce any appreciable shift in the I_ds_ versus V_g_ suggesting specific interactions of IL-6 on PASE conjugated aptamer surface gave rise to the change in electrical signal. Both Z axis and phase image in an Atomic Force Microscope (AFM) suggested unambiguous molecular interaction of the IL-6 on the nanotube-aptamer surface at 1 pg/mL concentration. The concentration of 1 pg falls below the diagnostic gray zone for cancer (2.3 pg-4 ng/mL), which is an indicator of early stage cancer. Thus, nanotube micro-arrays could potentially be developed for creating multiplexed assays involving cancer biomarker proteins and possibly circulating tumor cells all in a single assay using PASE functionalization protocol.

## 1. Introduction

Single wall carbon nanotubes are ideal candidates for label-free sensing; every atom is on the surface, hence their electronic properties are very sensitive to the surrounding charge environment [1]. Many reports have described carbon nanotube sensors for the detection of free proteins [2,3,4], DNA [5,6,7], viruses [8,9], and even cancer cells [10,11,12,13]. For many of these, the sensing mechanism is the gating effect of the target proteins as they bind to the antibodies, bringing them near the carbon nanotube (CNT) channels within the Debye length [14,15]. When using antibodies as recognition elements, the large size of the antibody can enable the target protein to interact outside the space charge layer or the Debye length of the sensor. This could reduce the sensitivity and specificity of the biosensor. Thus, antibody binding fragments [16] and aptamers [17] have been proposed for sensing very small concentrations of proteins. In the past nanotube-aptamer technology has been used to detect the binding of thrombin, IgE, and VEGF [18,19,20]. Researchers previously demonstrated IL-6 detection using antibody-nanotube systems with multiple label and amplification strategies [21,22,23]. For the first time, this study demonstrates label-free nanotube micro-array platforms using PASE conjugated aptamers for detection of IL-6 protein, an important cancer biomarker.

Interleukin-6 (IL-6) is a multi-functional cytokine characterized as a regulator of immune and inflammatory responses [24,25]. IL-6 plays a crucial role in tumor microenvironment regulation in a number of cancers, including breast, oral, prostate, and pancreatic cancers [26,27,28,29,30,31,32]. Higher levels of IL-6 in the blood of patients have been associated with advanced/metastatic cancers [26,33,34,35]. Overexpression of IL-6 receptor has also been shown in cervical cancer cells [36]. Such findings have increased the interest in development of anti-IL-6 therapeutics to target many of related diseases [37,38,39,40,41]. In this environment, an efficient, simple, and reliable diagnostics technique is of significant interest, and nanotube micro-arrays can help in sensitive detection and validation from multiple sensors in the same arrays. Having the ability to detect the levels of IL-6 in blood can help diagnose cancer at early stages. Further, the smaller arrays that have been developed for detection of cancer cells in buffy coats in the past [10] and more recently demonstrated larger arrays to capture spiked breast cancer cells in blood using the same PASE functionalization protocol [12,13] thus provide a unique opportunity to create multiplexed assays for the detection of proteins and capture of circulating tumor cells simultaneously all in one digital format. This may enable higher information content on the disease progression.

Detection of IL-6 is only applicable at low concentrations, 12–300 pg/mL, for practical clinical utility in metastatic cancers [34,42,43,44,45]. Electrochemical sensing of IL-6 has been reported using different methods, namely gold nanoparticles, carbon nanotube forest electrodes, and ferrocene loaded polyelectrolyte nanoparticles [21,22,23]. Detecting very small amounts of biomarkers using CNT-FETs has been a challenge due to the complication that the relative large size of antibodies creates in regards to Debye length. The effect on the mobile charge on the material is not realized if the target molecule is placed Debye length away from the surplus charge [46], which can be the case in the ionic environment that the CNT-FET biosensors operate in for biological applications with reference to the antibody size. To solve this issue aptamers have been used for biosensing of immunoglobin E (IgE) using CNT-FETs and showed improvement in sensor performance compared to an antibody coated nanotube channel under similar conditions [17]. Using aptamers, this study demonstrated the ability to detect IL-6 at concentrations as low as 1 pg/mL by using nanotube micro-arrays.

## 2. Results

**Figure 1:**
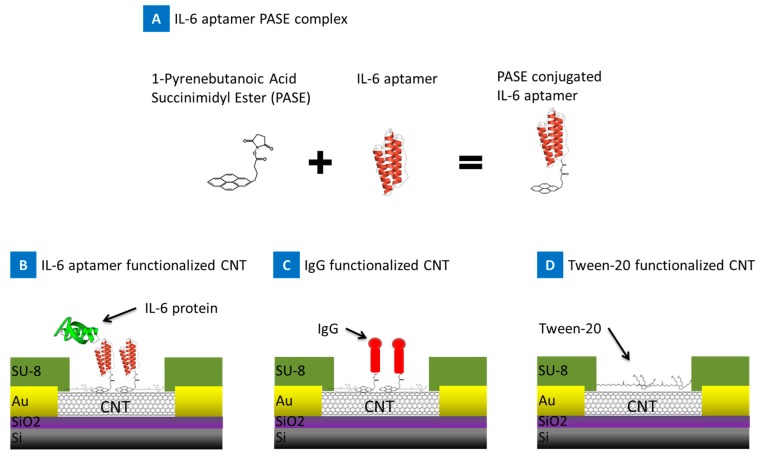
Schematic showing the reaction of 1-Pyrenebutanoic Acid Succinimidyl Ester (PASE) onto IL-6 aptamer forming the IL-6 aptamer PASE complex. Three groups of devices were prepared, Carbon Nanotube (CNT) device functionalized with (**B**) IL-6 aptamer PASE complex, positive control; (**C**) IgG, negative control; and (**D**) tween-20 blocking agent, negative control.

This paper demonstrates the detection of ultra-low levels of IL-6 protein (BioLegend, Cat.#: 575704) using PASE conjugated IL-6 aptamer functionalized on the nanotube channels. The RNA-aptamer specific to the IL-6 protein was used as the recognition element. The pyrene rings of the 1-pyrenebutanoic acid, succinimidyl ester (PASE) adsorb on to the sidewalls of the SWNT through π stacking [10,11,12,13]. The PASE conjugated IL-6 aptamers composite (Length: 32 nts, Base Pair Biotechnologies, Cat.#: ATW0077; 5′-NH_2_ modification) was adsorbed on to the side wall of the SWNT transistor to produce a stable nanotube-PASE-aptamer composite as presented in Figure 1a. The amine group on the PASE provides an attachment to the NH_2_ modified aptamers similar to past reports [17]. Two other variations of the devices were also prepared as negative controls: nonspecific antibody IgG-PASE functionalized Figure 1b and tween-20 blocked nanotubes Figure 1c.

**Figure 2:**
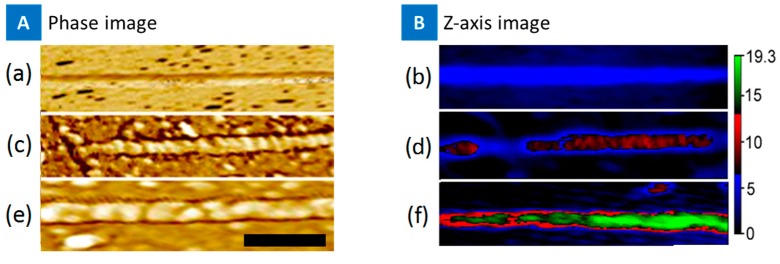
Atomic Force Microscope (AFM) image showing a phase image (**A**); and *Z*-Axis image (**B**), of bare CNT (**a**,**b**), functionalized CNT with IL-6 aptamer (**c**,**d**), and IL-6 protein binding to aptamer/CNT structure (**e**,**f**). Images were obtained using dry tapping mode (Nanosurf NaioAFM system). Scale bar = 100 nm.

Atomic Force Microscopy (AFM) was used to image the bare nanotubes, PASE-aptamer functionalized nanotube, and the binding of IL-6 on the functionalized nanotube surface. Figure 2 presents the *Z*-axis and the AFM phase image of bare nanotubes (blue), PASE-aptamer conjugate adsorption to the nanotube side wall in red, and the IL-6 binding in green. The phase image distinguishes the different proteins in two different devices, and the height was ~3 nm. The green color of the IL-6 binding shows almost complete coverage on the nanotube surface, suggesting the devices may have high sensitivity based on PASE-functionalization protocol.

**Figure 3:**
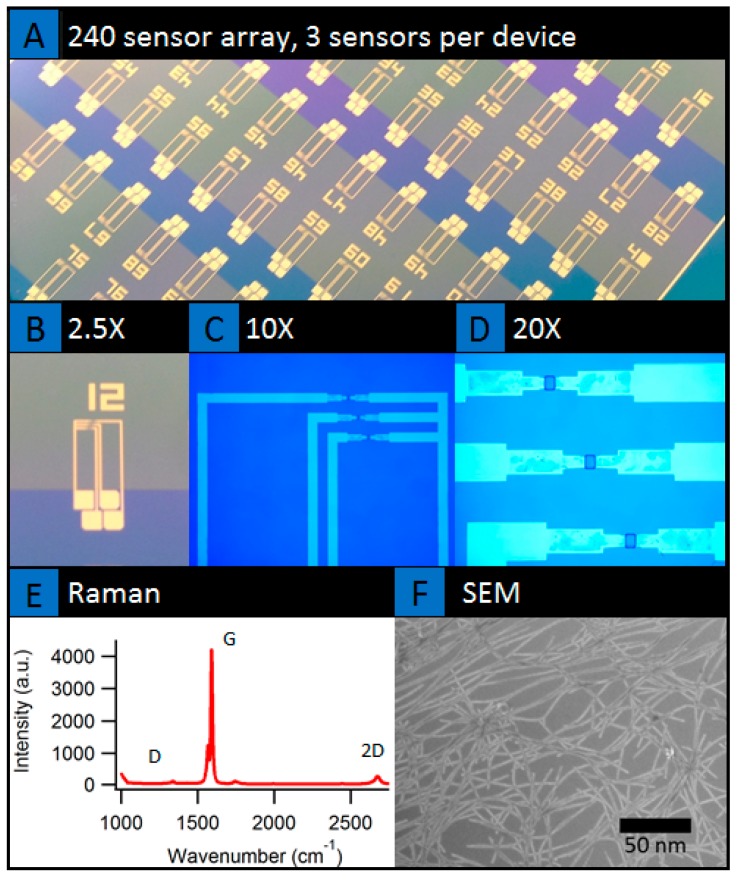
(**A**–**D**) Device characterization showing optical images of the sensor array platform at different magnification, (**E**,**F**) Raman characterization, and SEM (Scanning Electron Microscopy) image of the CNT film imbedded within the sensors respectively.

SWNT devices were fabricated using vacuum filtration of carbon nanotubes and device processing inside a clean room [12]. Figure 3 presents the wafer scale image of the 240 element array of nanotube network sensors. The sensor arrays were developed using a combination of vacuum filtration of carbon nanotube network film onto oxide coated wafers followed by multiple photo-lithography and reactive ion etching as presented in schematic (Figure 3b). Vacuum filtration is one of the preferred methods of making macroscopic and transparent networks of randomly oriented/highly aligned carbon nanotubes thin films and transistor devices. The film can be formed by using stock solutions of known concentrations. Vacuum filtration of SWNT suspension creates a concentration gradient due to the fluid velocity across the membrane [47]. With appropriate bulk solution concentration and fluid velocity, one can form either isotropic or highly oriented nanotube films [47]. While vacuum filtration has been used, in general, to make randomly oriented bucky papers, recent work by the authors has demonstrated the formation of isotropic-nematic transition of semiconducting nanotube films at ultra-low concentrations than what was thought to be achievable [47]. These semiconducting nanotube films are finding applications as thin-film transistors with high mobility and ON/OFF ratio’s [47], nanotube liquid crystal elastomer based light-driven actuators [48], label-free capture of cancer cells [12], and as sensors for cancer biomarkers as presented here. These films were low concentrations (3 µg) and most were isotropic, with the 2–4 µg samples partially transitioning into the nematic domains [47]. Mostly single layers laying on the substrate is shown in the SEM image of Figure 3f.

**Figure 4:**
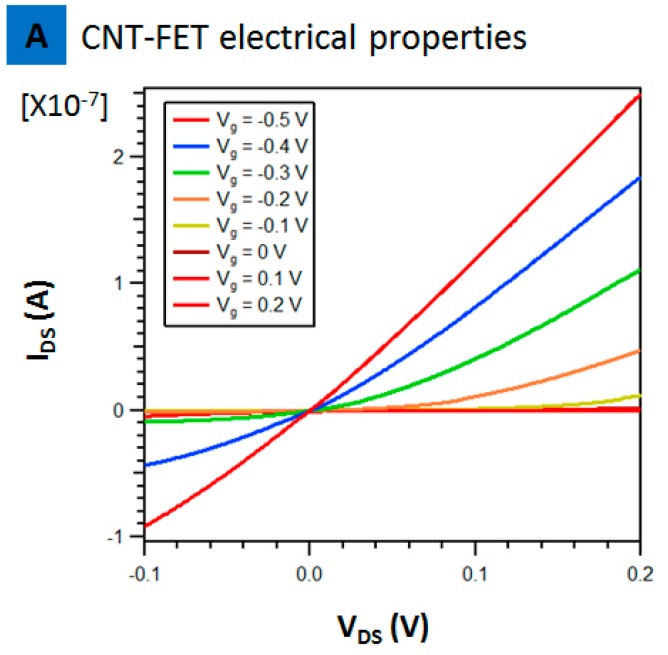
Electrical properties of CNT-FET devices before PASE-aptamer functionalization in phosphate buffer solution (1× PBS, pH 7.4).

Figure 4 presents the characteristics of a single array, CNT-FET, in buffer solution. The source to drain current (I_DS_) versus source to drain voltage (V_DS_) at different gate voltages is presented in Figure 4. The source to drain current decreased with increasing positive gate voltage. This suggests the device works as a p-type semiconductor in 1× PBS. No leakage was seen as at positive voltage the current is almost zero suggesting superior p-type transistor compared to past reports where there was still a significant drain current at zero and increasingly positive gate voltage [17]. This CNT-FET is also definitely superior compared to past reports on using CNT-FET with antibody fragments that used a mixture of metallic and semiconducting nanotubes resulting in poor on/off ratios [16]. This point is important and needs to be emphasized as the whole sensing principle relies on the charge introduced at the nanotube channels either reducing or increasing the conductance with introduction of IL-6 protein. Leakage currents or poor on/off ratios can thus affect the sensing reliability if one needs to detect such ultra-low concentrations. Thus, at V_g_ = 0 V, the increase or decrease in conductance with introduction of protein is the result of binding alone and not the result of the currents in the substrate.

**Figure 5:**
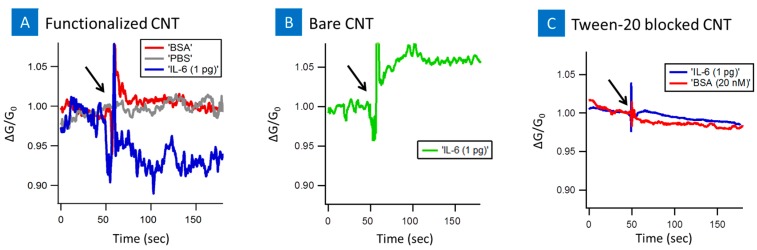
Sensor response of the CNT-biosensor at source-drain bias of 0.1 V and at the gate bias of 0 V. (**A**) indicates the response after the introduction of 20 nM BSA (red line), 1 mM MgCL_2_ 1× PBS (gray line), 1 pg IL-6 target protein (blue line) to functionalized CNT-biosensor and (**B**) 1 pg IL-6 target protein to bare unfunctionalized CNT-biosensor (green line); (**C**) Response of tween-20 blocked CNT-biosensor to IL-6 protein (blue line), and BSA (red line). Arrow indicates the point of sample injection.

Figure 5 presents the real time sensing of IL-6 protein on the PASE-aptamer functionalized and tween-20 blocked nanotube. First, a 5 μL droplet of 1 mM MgCl_2_ 1× PBS was placed on the functionalized device. The change in conductance was recorded, followed by introduction of 1 pg/mL of IL-6. As soon as IL-6 was introduced to the PASE-aptamer functionalized devices, the conductance decreased and reached a saturation value as shown in blue color line of Figure 5a. Introduction of plain PBS or BSA on the functionalized surface showed little/no change and is shown in red and grey line colors. Additionally, devices with tween-20 blocked nanotubes showed no significant change after introduction of IL-6 protein or BSA (Figure 5c). Figure 5b presents the time dependence of the normalized conductance on introducing 1 pg/mL IL-6 to the bare unfunctionalized nanotube channel. The increase in conductance of the CNT-FET was clearly observed after introduction of 1 pg of IL-6 onto bare CNT-FET without aptamers. Since IL-6 were non-specifically interacted with the bare CNT channels, the entire molecule must be placed inside the Debye length. IL-6s were negatively charged under our experimental conditions (pH 7.4), which was relatively higher than the isoelectronic point of IL-6 (pH(I) ~6.96). The increase in conductance is therefore expected as the negative charge on the IL-6 is equivalent to applying a negative gate voltage [17].

**Figure 6:**
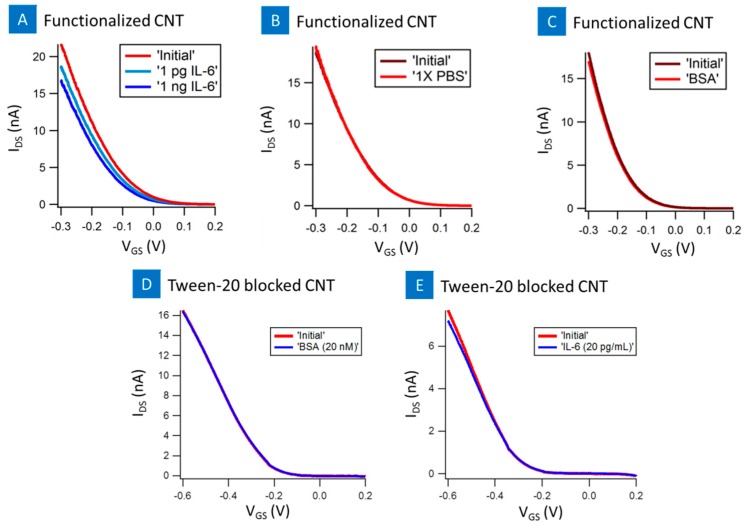
Before and after I_DS_-V_GS_ characterization of IL-6 aptamer functionalized CNT-biosensor with (**A**) blank 1 mM MgCL_2_ 1× PBS samples; (**B**) 20 nM BSA; and (**C**) 1 pg and 1 ng IL-6 protein injection. I_DS_-V_GS_ response of tween-20 blocked CNT-biosensor to BSA (**D**); and IL-6 protein (**E**).

Figure 6 presents the transistor device characteristics for PASE-anti-aptamer functionalized nanotube FET. All the devices were p-type with average *I*_on_/*I*_off_ of ~180 and the nanotubes were semiconducting carbon nanotubes [12]. On introduction of PBS as well as BSA controls on the functionalized nanotube surface there was no appreciable shift in the I-V_g_ curve. However, on introduction of 1 pg/mL of IL-6 and 1 ng/mL of IL-6 on two different devices with similar initial characteristics, there was a change in the I-V_g_ curve. The I-V_g_ curve shifted down with increasing concentration suggesting positively charges by IL-6 decreased the conductance of the nanotube device. This is equivalent to applying a positive gate voltage and has been seen in previous reports [17]. The conjugated negatively charged part of the IL-6-aptamers was placed inside the electrical double layer in the buffer solution, local positive charge in IL-6 molecules was detected as a decrease in conductance [17]. Additionally, tween-20 blocked devices showed no significant shift in the I-V_g_ curve as a result of introduction of IL-6 or BSA samples, Figure 6d,e. The specificity of the device is observed from the controls, PBS and BSA control samples and tween-20 blocked device, which showed no response to the nanotube sensor. Thus, the results suggest that non-specific binding was successfully suppressed using aptamer functionalized nanotube surface.

**Figure 7:**
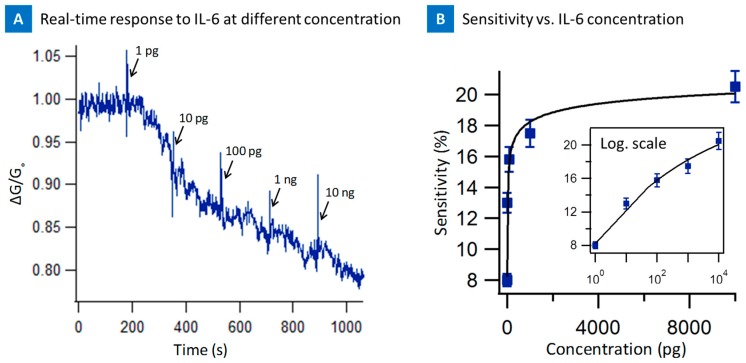
(**A**) Time dependence of normalized source-drain current of the CNT-biosensor at the source-drain bias of 0.1 V and at gate bias of 0 V after the introduction of IL-6 target protein at various concentrations onto the IL-6 aptamer functionalized CNT-biosensor. Arrows indicate the points of IL-6 target protein injection; (**B**) The Sensitivity of IL-6 aptamer CNT-biosensor as a function of IL-6 protein concentration (1 pg to 10 ng /mL). The inner box demonstrates the data in logarithmic scale.

Figure 7a presents the real time response of the nanotube sensor from 1 pg/mL to 10 ng/mL of the Il-6. Each concentration of the IL-6 was seen with a characteristic spike as well as decrease in conductance as seen in all the experiments before. The change in normalized conductance ratio of the device went from 1.0 to less than 0.80 suggesting, interaction of the IL-6 and screening of the nanotube surface by IL-6 due to the interaction with IL-6-PASE aptamer. The device saturated beyond 100 ng suggesting all the binding sites were depleted above these concentrations on the device and thus suggesting a saturated response. Figure 7b presents the sensitivity as a function of concentration. The linear range at low concentration suggests sensitivity for 1–100 pg, which could be useful for biomarker testing.

**Figure 8:**
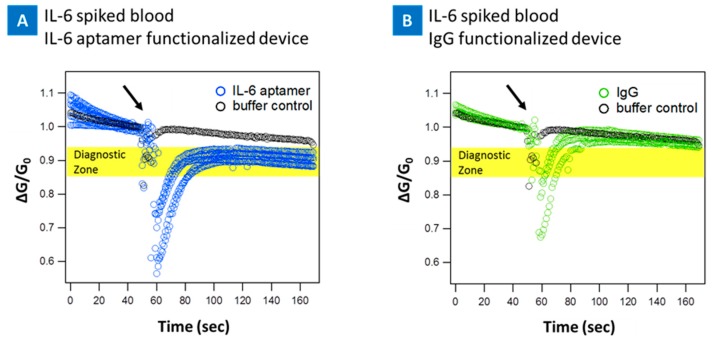
Blood experiment results. Response of the CNT-biosensor at source-drain bias of 0.1 V and at the gate bias of 0 V. IL-6 spiked blood samples were tested on two different CNT-biosensor surfaces, IL-6 aptamer (**A**), and IgG (**B**) functionalized surfaces. The response of the IL-6 aptamer functionalized devices to control buffer, 1 mM MgCl_2_ 1× PBS, is presented in each panel as a reference point (black marks). (**A**) Shows the respond of the IL-6 aptamer functionalized device to IL-6 protein spiked blood (10 pg/mL), ~8–13% drop in the current (diagnostic zone); (**B**) Presents the response to IgG functionalized CNT-biosensor, negative control surface, to IL-6 spiked blood showing no significant change in signal. Arrows indicate the points of IL-6 spiked blood injection, at 60 s.

**Figure 9:**
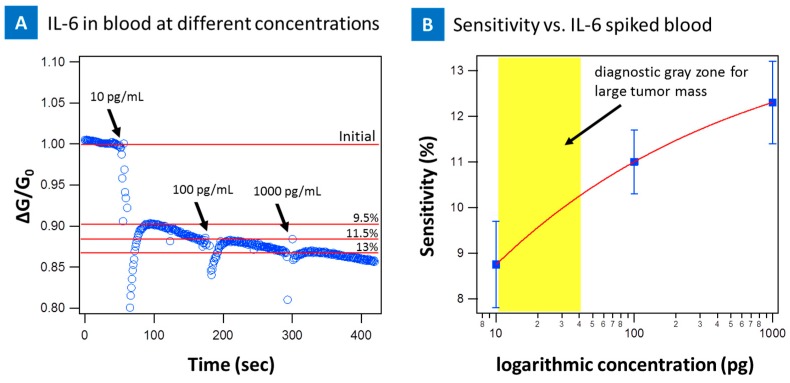
(**A**) Time dependence of normalized source-drain current of the CNT-biosensor at the source-drain bias of 0.1 V and at gate bias of 0 V after the introduction of IL-6 spiked blood sample at various concentrations onto the IL-6 aptamer functionalized CNT-biosensor. Arrows indicate the points of IL-6 spiked blood injection; (**B**) The Sensitivity of IL-6 aptamer CNT-biosensor as a function of IL-6 protein concentration in blood sample (10 pg to 1 ng /mL) in logarithmic scale. Diagnostic gray zone for large tumor mass at ~12–40 pg/mL is highlighted in yellow which is higher than our sensor’s lower limit sensing threshold at 10 pg/mL.

To further investigate the feasibility of the CNT micro-array sensors in clinical setting with patient samples, a series of tests was done by spiking fresh blood with IL-6 protein and introducing the sample to the devices. The real time sensing of IL-6 protein spiked into blood at 10 pg/mL on the PASE-aptamer functionalized, Figure 8a, and control IgG-PASE functionalized Figure 8b nanotube device are presented. First, a 5 μL droplet of 1 mM MgCl_2_ 1× PBS was placed on the functionalized device. The change in conductance was recorded, followed by introduction of 10 pg/mL of IL-6 spiked blood. As soon as IL-6 spiked blood was introduced to the PASE-aptamer functionalized devices, the conductance decreased and reached a saturation value as shown in Figure 8a for five different devices. On the other hand, there was no significant change in signal when the same sample was introduced to IgG functionalized device (Figure 8b). For the blood samples, the sensitivity of device was decreased to 10 pg/mL as a result of the complexity of blood sample environment. Figure 9a presents the real time response of the nanotube sensor from 10 pg/mL to 1 ng/mL of the IL-6 spiked blood. The change in signal is less significant above 10 pg/mL concentration compared to buffer spiked samples. This suggests the devices saturate much quicker as a result of the blood environment and completely saturate beyond 1 ng suggesting all the binding sites were depleted above these concentrations on the device and thus a saturated response. Figure 9b presents the sensitivity as a function of concentration. 10 pg/mL sensitivity for blood samples falls within the diagnostic gray zone for cancer (1–100 pg/mL) and therefor can still be clinically relevant [49].

## 3. Discussion and Conclusions

The ability to detect IL-6 at low concentrations in serum can have implications in the clinic. IL-6 levels in serum >12 pg/mL have been correlated with large tumor size and elevated Serum-C Reactive Protein (CRP) levels for colorectal cancers [50]. Patients with undetectable levels of IL-6 in the serum had a mean CRP level of 27 mg/L, whereas it was 43 mg/L for patients with detectable IL-6 levels (~300 pg/mL) [26,33,34,35]. Patients with IL-6 levels >300 pg/mL exhibited CRP levels of 117 mg/L [26,33,34,35]. IL-6 is detected due to the secretion of IL-6 from the cancer cells into the blood stream and thus very low detection thresholds can be indicators of cancer development and can lead to life saving measures.

In the past the gold nanoparticle immunosensor gave a detection limit (DL) of 10 pg /mL of IL-6 in 10 μL calf serum [21]. The sensitivity of SWNT forest immunosensor was 0.5 pg/mL for the same assay protocol but this assay uses multi-label detection technique and is inferior to label free techniques [21]. Our nanotube-FET with 1 pg/mL in buffer solution and 10 pg/mL in blood sensitivity is thus capable of achieving 10 pg/mL sensitivity in serum. However, to determine the final specificity of IL-6 biosensing platforms, including our micro-arrays, other cytokines such as G-CSF, IL-4, TNF-α and IFN-γ must be tested in order to address exclusion of possible ambiguous recognition. These are the next steps in being able to use these micro-arrays in unaltered blood that can detect both levels of IL-6 and circulating tumor cells. The big unanswered question is, is there a correlation between levels of IL-6 and CTCs in blood that could be indicative of the metastatic condition? Past reports have shown that increased level of serum IL-6 in some patients with lung cancer has been reported previously and shown to be part of an inflammatory response [51]. The nanotube micro-arrays with the PASE functionalization protocol thus paves the way to detect these two important targets for diagnosis, treatment and progression of cancer.

To conclude, this study presented an approach to detect an important cancer biomarker, namely IL-6 using semiconducting nanotube channels. IL-6 in the level of 1 pg/mL to 10 ng/mL was detected successfully in both buffer and blood samples using a PASE conjugated IL-6 aptamer as the molecular recognition element. The nanotube micro-array is thus capable of capturing cancer cells using antibodies and detecting IL-6 using aptamers. An assay that could detect both proteins and capture cells simultaneously can lead to an understanding between the serum levels of IL-6, CRP levels and the number of CTCs in blood.

## 4. Materials and Methods

### 4.1. Device Fabrication

The sensors are a three-terminal design. A carbon nanotube network connects the source and drain Ni/Au electrodes and the electrodes are covered by an insulating layer of SU8 photopolymer. Gate voltage is applied through an external Ag/AgCl reference electrode.

The first step in the process is assembling the nanotube network. A 99% weight IsoNanotubes-Semiconducting single wall carbon nanotube mixture was purchased from NanoIntegris (c/o Raymor Industries, 3765 La Verendrye, Boisbriand, QC, Canada). Nanotubes are listed at 1.2–1.7 nm diameter and 300 nm to 5 microns length. Nanotubes were suspended in surfactant solution at 1 mg/100 mL when received. 300 µL of the stock solution, 3 µg of CNT, were aspirated and then diluted to 3 μg/100 mL solution of DI water and sodium dodecyl Sulfate (SDS) (Sigma-Aldrich, St. Louis, MO, USA; Cat.#: 436143).

The 100 mL solution was vacuum filtered over a cellulose membrane, 0.05 μm pore size (Millipore, Billerica, MA, USA; Cat.#: VMWP09025). This method self-regulates the deposition rate of nanotubes on the membrane to produce an evenly distributed network. The network is then pressed onto a dry oxidized (300 nm thickness) silicon wafer for 30 min. Next, the wafer is transferred to an acetone vapor bath which dissolves the membrane.

Patterning of the nanotube film and electrode and insulating layer fabrication are done by photolithography in the cleanroom. SC1813 photoresist is used to mask the nanotube film areas needed for the sensor elements. Exposed nanotubes are etched away in a March reactive ion etcher for 90 s at 200 W power with oxygen plasma. SC1827 photoresist is used to mask the electrode pattern. Electrodes consist of a 15 nm Ni adhesion layer and a 90 nm Au layer. They are deposited by sputtering in a Leskar PVD 75 system, 300 W DC power. Last, the sensors are covered with SU8–2005, a 5 μm thick photopolymer layer. A window over each of the nanotube sensor elements is developed but the electrodes remain insulated beneath the SU8, Figure 1.

### 4.2. Device Functionalization

CNT biosensors were functionalized with 1-Pyrenebutanoic Acid Succinimidyl Ester (PASE) conjugated IL-6 aptamers (Base Pair biotechnologies, Pearland, TX, USA; Cat.#: ATW0077) at 20 μg/mL in 1 mM MgCL_2_ 1× PBS buffer solution for 2 h at room temperature. For the IgG antibody, functionalization devices were incubated in the PASE (AnaSpec, Freemont, CA, USA; Cat. No. 81238), dissolved in methanol at 1 mM, solution for 2 h at room temperature, and then rinsed with methanol and dried using a nitrogen air gun and then then incubated in IgG (EMD Millipore, Billerica, MA, USA; Cat. No. 411550), 20 μg mL^−1^ in 1 mM MgCl_2_ 1× PBS, for 2 h at room temperature. Devices were rinsed three times in 1 mM MgCl_2_ 1× PBS and then were functionalized with tween20 to block unfunctionalized/bare nanotube sidewalls to minimize non-specific interactions. Devices were incubated with 0.5% *v*-*v* Tween20 suspended in buffer solution (1 mM MgCL_2_ 1× PBS) for 2 h at room temperature. After incubation, devices were rinsed in 1 mM MgCl_2_ 1× PBS solution, and then incubated in a droplet of 1 mM MgCl_2_ 1× PBS overnight in a humid chamber at 4 °C for testing. Tween-20 blocked devices were solely functionalized with Tween-20 for 2 h as described above.

### 4.3. Blood Sample Preparation

50 μL of blood was collected from a volunteer subject using a lancing device for each set of tests. The collected blood sample was transferred into a vial and diluted 1:4 with 1 mM MgCl_2_ 1× PBS buffer solution containing IL-6 protein to create IL-6 spiked samples accordingly for 10, 100, and 1000 pg/mL final concentrations. The prepared spiked blood samples were immediately used for testing. Plain blood samples were prepared in the same fashion and diluted in buffer solution 1:4.

### 4.4. Testing

Bare non-functionalized, IL-6 aptamer functionalized, and tween-20 functionalized “blocked” devices were prepared. All three batches of devices were tested with 2 μL droplets of 1 mM MgCl_2_ 1× PBS (blank buffer solution), BSA (20 nM in buffer solution) and IL-6 target protein (1 pg to 10 ng/mL in buffer solution) individually.

For the blood experiments, two batches of devices were also prepared: IL-6 aptamer functionalized (positive control) and IgG functionalized (negative control). Both batches of devices were tested with 2 μL droplets of IL-6 spiked blood samples (10 pg to 1 ng/mL) individually.

100 mV source-drain bias and 0 V gate bias applied across the sensor and the source drain current, I_ds_, was monitored vs time. Two μL droplet of sample solution, 1 mM MgCl_2_ 1× PBS, BSA, or IL-6 protein was pipetted directly into the standing 5 μL 1 mM MgCl_2_ 1× PBS droplet on top of the devices. The change in current flow was monitored for the next three minutes. Devices were tested on a signatone probe station platform using an Agilent 4156C semiconductor parameter analyzer with a Lab View Software. The entire probe station assembly is placed on an optical table that is vibration isolated using air on all four legs. A metal box covers the entire assembly to avoid electromagnetic interference. The probes are connected to the parameter analyzer using a triaxial cable that is EM shielded. Throughout the testing the devices were maintained inside a humid chamber to prevent evaporation of the sample droplet.
